# VceC Mediated IRE1 Pathway and Inhibited CHOP-induced Apoptosis to Support *Brucella* Replication in Goat Trophoblast Cells

**DOI:** 10.3390/ijms20174104

**Published:** 2019-08-22

**Authors:** Feijie Zhi, Dong Zhou, Furong Bai, Junmei Li, Caixia Xiang, Guangdong Zhang, Yaping Jin, Aihua Wang

**Affiliations:** 1 Key Laboratory of Animal Biotechnology of the Ministry of Agriculture, Northwest A&F University, Yangling 712100, China; 2 College of Veterinary Medicine, Northwest A&F University, Yangling 712100, China

**Keywords:** *Brucella suis* S2, type IV secretion system, VceC, goat trophoblast cells, apoptosis, endoplasmic reticulum stress, unfold protein response

## Abstract

The effectors of the type IV secretion system (T4SS) of bacteria play important roles in mediating bacterial intracellular proliferation and manipulating host-related pathway responses to bacterial infection. *Brucella* Spp. inhibit the apoptosis of host cells to benefit their own intracellular proliferation. However, the underlying mechanisms between T4SS effectors and *Brucella*-inhibited apoptosis in goat trophoblast cells remain unclear. Here, based on *Brucella suis* vaccine strain 2, the VceC was deleted by allelic exchange. We show that ΔVceC was able to infect and proliferate to high titers in goat trophoblast cells (GTCs) and increase C/EBP-homologous protein (CHOP)-mediated apoptosis. GRP78 expression decreased upon ΔVceC infection. In addition, we discovered that the inositolrequiring enzyme 1 (IRE1) pathway was inhibited in this process. Changing endoplasmic reticulum (ER) stress affected *Brucella* intracellular replication in GTCs. The replication of ΔVceC was more sensitive under the different ERstress conditions in the GTC line after treatment with ER stress inhibitors 4 phenyl butyric acid (4-PBA) or ER stress activator Tm. Together, our findings show that VceC has a protective effect on the intracellular persistence of *Brucella* infection, and inhibits ER stress-induced apoptosis in the CHOP pathway. The present work provides new insights for understanding the mechanism of VceC in the establishment of chronic *Brucella* infection.

## 1. Introduction

Brucellosis is a zoonotic infectious disease caused by bacteria of the genus *Brucella*. The infection affects more than 500,000 people across the world, a number that may be higher in agricultural communities worldwide [1,2]. *Brucella*, a Gram-negative and facultative intracellular bacterium, mainly parasitizes in phagocytic cells, such as macrophages and trophoblast cells [3,4]. The pathogenicity of *Brucella* is due to its ability to adapt to the environmental conditions encountered in its intracellular replicative niche including low levels of nutrients and oxygen, acidic pH, and reactive oxygen intermediates [5]. To date, no vaccine can be safely and effectively used to prevent human brucellosis, and the disease in human is difficult to treat with antibiotics [6]. Because of the *Brucella* characteristics, it could be used as a bioweapon [7]. The *Brucella* vaccines, strain 19 and RB51, are effective in controlling brucellosis in animal [8]. *Brucella suis* vaccine strain 2 (*B. suis* S2), a naturally attenuated variant in china, was isolated from the embryo of an aborted sow in 1952 by the researchers of China Institute of Veterinary Drug Control and is most extensively used for the prevention and control of brucellosis in sheep, goats, cattle, and other domestic animals [9]. However, these vaccines have numerous drawbacks, including interference with diagnostic tests, pathogenicity for humans, potential to cause abortion in pregnant animals, and so on. Therefore, it is critical to understand the molecular mechanisms of *Brucella* intracellular survival and proliferation during infection for preventing brucellosis and developing vaccines.

The type IV secretion system (T4SS) is essential for persistent *Brucella* infection, since T4SS mutants are incapable of intracellular survival and replication in phagocytic cells and attenuated in a mouse model of infection [10]. T4SS injects *Brucella* effector proteins from the bacterium into the host-cell cytosol to impact cellular homeostasis and normal physiology. De Jong et al. [11] provide first direct evidence that effector protein VceC is conserved in all sequenced *Brucella* genomes and is translocated into cells by the *Brucella* T4SS. The translocated VceC results in a cytotoxic effect on macrophages. VceC translocates to the endoplasmic reticulum (ER) where it binds the GRP78 and induces an IRE1α-dependent inflammation [12]. Keestra-Gounder et al. [13] suggest that VceC can trigger ER stress, contributing to abortion during *B. abortus* infection in mice.

In animal primary hosts, *Brucella* have a particular tropism for the reproductive system, often leading to abortion in pregnant female animals. Because of the presence of high *Brucella* loads within placental trophoblast cells, the infection ultimately results in disruption of the placenta and infection of the fetus [14]. Therefore, trophoblast cells are a primary cellular target for the efficient survival and proliferation of *Brucella* in the natural host. However, the molecular mechanisms of the *Brucella* infectious process in goat trophoblast cells (GTCs) remain unclear. Once inside the host cells, *Brucella* in turn interact with the early and late endosomes, ER, and autophagy-like vacuoles, resulting in the completion of the intracellular lifecycle of *Brucella* and cell-to-cell spreading [4,5]. *Brucella* require fusion with the ER for survival, establishing a proliferation niche, and multiplication within host cells [15]. The ER fusion dramatically restructures the ER and disrupts ER homeostasis, leading to a condition known as ER stress [16]. To maintain ER homeostasis, the unfolded protein response (UPR) is induced, especially by the inositol-requiring enzyme 1 (IRE1) pathway, which promotes *Brucella* intracellular survival and proliferation in macrophages or GTCs [13,17]. In response to ER stress, the binding immunoglobulin protein (Bip, also known as GRP78) is recruited away from three sensors that are located in the ER membrane to assist in refolding proteins within the ER, resulting in activation of the UPR signaling pathway [18]. However, when persistent or excessive ER stress exceeds the ability of the UPR to manage misfolded and unfolded proteins, the UPR switches from an adaptive pathway to one that induces cell death [18]. UPR-mediated apoptosis is a new apoptosis signaling pathway, and one of the most significant activations of this pathway is induced by CHOP [19]. The manipulation of host cell death is a critical strategy of *Brucella* to maintain dissemination and intracellular persistence. The VceC also mediates the cytotoxicity effect by translocation of this effector protein into macrophages resulting in lysis of the host cells [11]. However, the interaction of VceC and *B. suis* S2-induced apoptosis mediated by ER stress in GTCs has scarcely been studied.

Here, we constructed a *B. suis* S2 T4SS effector protein VceC deletion mutant and investigated the mechanisms of VceC on *B. suis* S2-induced apoptosis in GTCs. Our results showed that the VceC mutant increased CHOP expression, interacted with GRP78, and mediated IER1 pathway of UPR to promoted apoptosis in GTCs. The replication of the VceC mutant was more sensitive under the different ER stress conditions after treatment with ER stress inhibitors or inducers in the GTC line. These findings demonstrate that VceC is a vital *Brucella* virulence by activating ER stress and further manipulating UPR to inhibit GTC apoptosis during *Brucella* infection.

## 2. Results

### 2.1. Compare to VceC Amino Acid Sequences Derived from Different Brucella Strains

**Figure 1:**
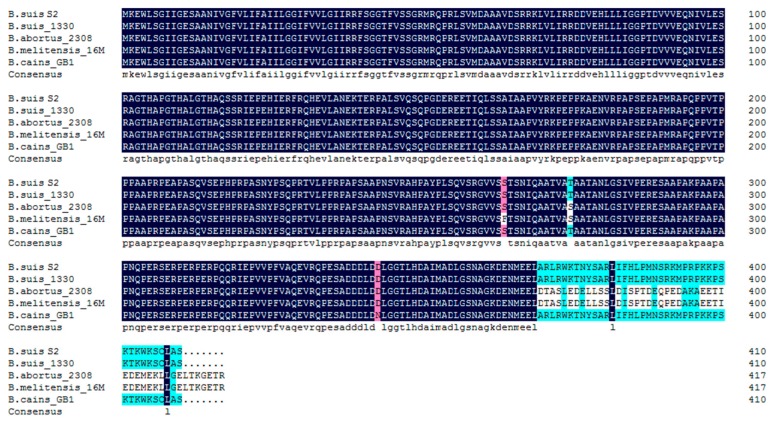
Alignment of VceC nucleotide sequences (**A**) and amino acid sequences (**B**) for *Brucella suis* S2 (BSS2_I1011), *B. suis* 1330 (BR1038), *B. abortus* 2308 (BAB1_1058), *B. melitensis* 16M (BEMI0948), and *B. cains* GB1 (C6Y57_05925). Amino acid differences are shaded in red and green.

VceC is present in all sequenced *Brucella* strains, including *B. suis* S2 (BSS2_I1011), *B. suis* 1330 (BR1038), *B. abortus* 2308 (BAB1_1058), *B. melitensis* 16M (BEMI0948), and *B. cains* GB1 (C6Y57_05925) (Figure 1). VceC amino acid sequences were compared and analyzed based on the above strains. The VceC protein of *B. suis* S2 contained 410 amino acids, with a proline rich central domain. An N-terminal region of approximately 260 amino acids was conserved in proteins of the above strains. A 1 bp missed in *B. suis* and *B. cains* GB1 *vceC* led to a frameshift in the C-termini of *B. suis* and *B. canis* GB1. VceC protein had one amino acid change (Ser-275) in *B. abortus* 2308, one amino acid change (Asn-343) in *B. cains* GB1, and two amino acid changes (Phe-264 and Ser-275) in *B. melitensis* 16M, when compared with *B. suis* S2.

### 2.2. Mutant Strains ΔVceC Intracellular Survival in GTCs

**Figure 2:**
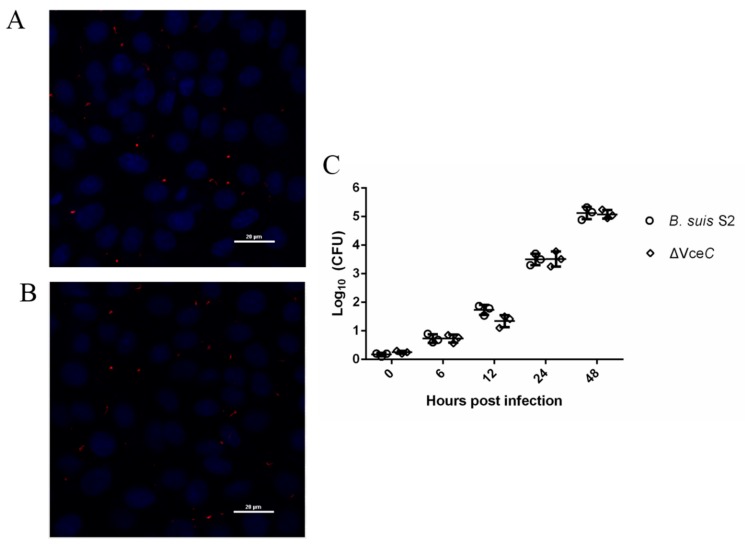
Infection and proliferation of *B. suis* S2 and ΔVceC in goat trophoblast cells (GTCs). (**A**,**B**) Representative confocal micrographs of GTCs infected with *B. suis* S2 (**A**, red) and ΔVceC (**B**, red) at 24 h post infection. The data shown are representative of three independent experiments. (**C**) Intracellular survival in GTCs of wild type (*B. suis* S2, circle) and the mutant obtained (ΔVceC, square). The results are expressed as the means ± standard deviation from three independent experiments at each time point.

The VceC gene deletion mutant based on *B. suis* S2 was successfully constructed. Trophoblast cells were a primary cellular target for *Brucella* in the natural host. Our previous study demonstrated *B. suis* S2 was able to infect GTCs cultured in vitro [17] To evaluate the bacterial adherence and intracellular survival in GTCs, the number of CFUs was counted following infection with *B. suis* S2 and the mutant strains ΔVceC at 100 multiplicity of infection (MOI). The bacterial adherence of ΔVceC was not significantly different compared to *B. suis* S2 strains (data not shown). We demonstrated *B. suis* S2 (Figure 2A) and ΔVceC (Figure 2B) were able to infect GTCs cultured in vitro. To determine whether deletion of VceC affected the intracellular survival of *Brucella*, the number of CFUs was counted following infection with *B. suis* S2 and ΔVceC at different times. The results indicated the intracellular survival of *B. suis* S2 and ΔVceC occurred in a time-dependent manner. However, the bacterial intracellular survival of ΔVceC was not significantly different (Figure 2C).

### 2.3. Effect of ΔVceC on GTC Apoptosis

To evaluate whether the deletion of VceC affected the apoptosis level of GTCs infected by *B. suis* S2, we assessed the effect of apoptosis by flow cytometry in combination with Annexin V/PI double staining. When the cells were infected with ΔVceC for 12 h and 48 h, the average apoptosis of only Annexin V-positive cells (early apoptosis cells) increased significantly, reaching approximately 3.04 ± 0.10% and 8.96 ± 0.99%, respectively (Table 1 and Table 2 and Supplementary Figure S1). However, the average apoptosis of early apoptosis cells to *B. suis* S2 was approximately 1.77 ± 0.23% and 5.15 ± 2.46%, respectively (Table 1 and Table 2 and Supplementary Figure S1). The results demonstrated that ΔVceC increased the proportion of early apoptosis in GTCs at 12 h and 48 h post infection.

**Figure 3:**
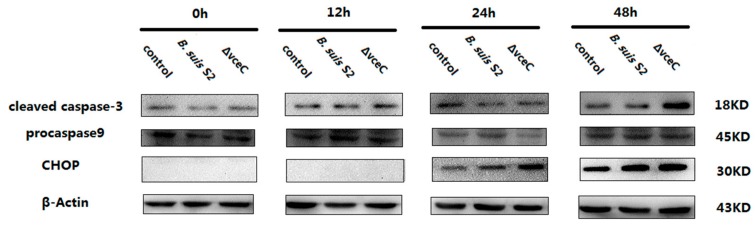
Effect of deletion of VceC on apoptosis of *Brucella* following infection of GTCs. GTCs were infected with 100 MOI of *B. suis* S2 and ΔVceC for 0 h, 12 h, 24 h, and 48 h, lysed, and subjected to Western blot analysis to detect the expression of apoptosis-related cleaved caspase-3, caspase-9, and CHOP proteins. The data shown are representative of three independent experiments.

Because ΔVceC infection increased cells’ early apoptosis, we measured cleaved caspase-3 (the marker of apoptosis), procaspase-9 (the marker of mitochondrion-induced cell death), and CHOP (the marker of ER stress-induced cell death) expression by Western blot at different times (Figure 3). At 48 h, cleaved caspase-3 expression was enhanced after ΔVceC infection compared with the *B. suis* S2 infected group. However, cleaved caspase-3 expression was not significantly different at 0, 12, and 24 h among the uninfected group, ΔVceC-infected group, and *B. suis* S2-infected group. Caspase-9 expression was also not significantly different at 0, 12, 24, and 48 h among the uninfected group, ΔVceC-infected group, and *B. suis* S2 infection group. Furthermore, CHOP expression was increased at 24 and 48 h after ΔVceC infection compared with the *B. suis* S2 infection group, but no significant difference was found at 0 and 12 h. According to our Western blot analysis, cleaved caspase-3 expression at 48 h, as well as CHOP expression at 24 h and 48 h, was more strongly induced in the ΔVceC infection group than in the *B. suis* S2 infection group. The results suggested VceC is involved in *Brucella*-mediated apoptosis through the UPR signaling pathway.

### 2.4. Deletion of VceC Decreases Brucella-Mediated ER Stress

**Figure 4:**
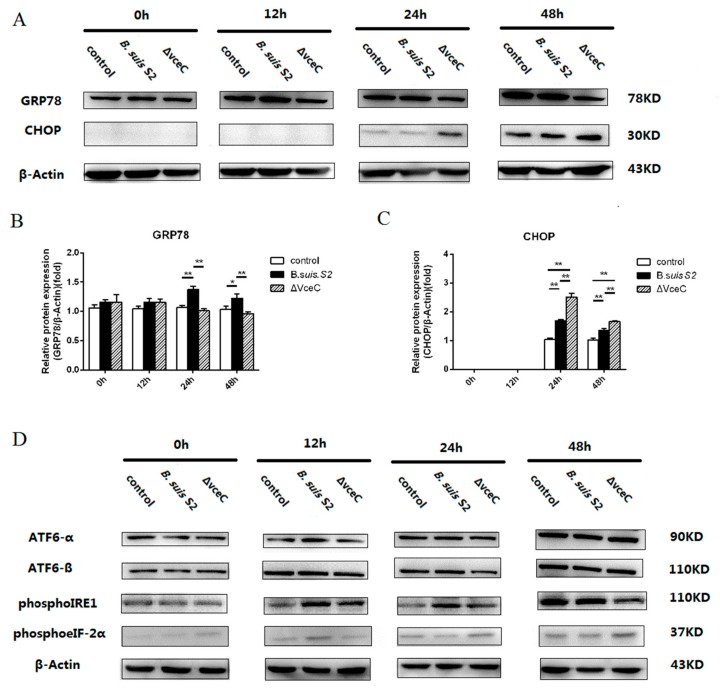
The unfolded protein response (UPR) pathway is induced by *B. suis* S2 and ΔVceC. (**A**) GTCs were infected with 100 MOI of *B. suis* S2 and ΔVceC for 0 h, 12 h, 24 h, and 48 h, and the untreated group was used as a negative control, followed by lysis and detection of GRP78 and CHOP protein expression by Western blot. The data shown are representative of three independent experiments. (**B**,**C**) Quantification of band intensities from three independent results was determined by densitometric analysis. Data represent the mean ± standard deviation from three independent experiments at each time point.* *p* < 0.05; ** *p* < 0.01 (**D**) GTCs were infected with 100 MOI of *B. suis* S2 and ΔVceC for 0, 12, 24, and 48 h, and the untreated group was used as a negative control, followed by lysis and detection of ATF6-α, ATF6-β, phosphoIRE1, and phosphoeIF-2α protein expression by Western blot. The data shown are representative of three independent experiments.

UPR is a cytoprotective response that is aimed at monitoring the survival and proliferation of intracellular pathogens. To more directly examine ER stress after ΔVceC infection in GTCs, we examined the expression of GRP78 in ΔVceC infection GTCs. GRP78 expression was increased at 24 and 48 h in the *Brucella*-infected group compared to the uninfected group and was decreased at 24 and 48 h in the ΔVceC-infected group compared to the *Brucella*-infected group, but no significant difference was found at 0 and 12 h among all groups (Figure 4A–C). The results indicate the deletion of VceC changes *Brucella*-mediated ER stress.

To investigate UPR induction during ΔVceC infection, GTCs were infected with *B. suis* S2 or ΔVceC, and the activation of three UPR sensors was analyzed by Western blot. ATF6-α, ATF6-β, and phosphoeIF-2α (downstream protein of PERK branch) were not significantly different in all groups (Figure 4D). However, PhosphoIRE1 expression was decreased at 12, 24, and 48 h in the ΔVceC infection group compared to the *Brucella* infection group (Figure 4D). We conclude that VceC is involved with *Brucella*-mediated ER stress by IRE1 branch activation of UPR.

### 2.5. The Replication of ΔVceC Was More Sensitive after Changing ER Stress in GTCs

**Figure 5:**
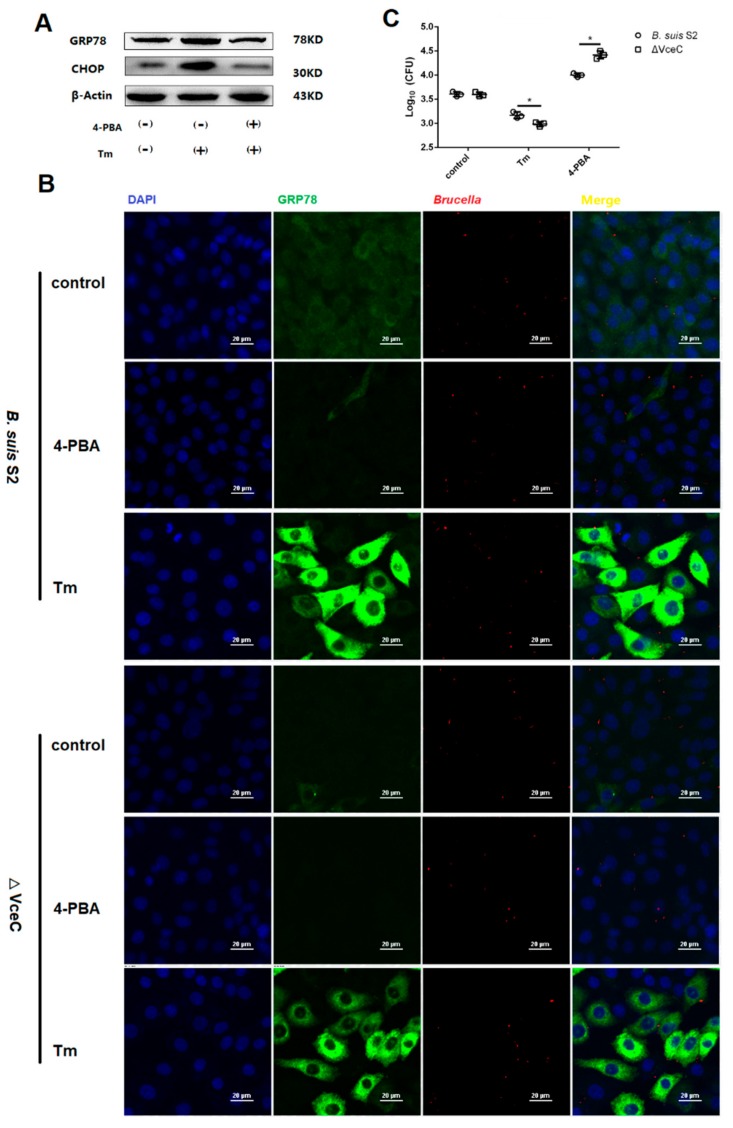
Intracellular survival of *B. suis* S2 and ΔVceC by changing endoplasmic reticulum (ER) stress. (**A**) The UPR marker GRP78 and CHOP were analyzed using Western blot by treating with 0.5 µg/mL Tm or 1 µM 4-PBA in GTCs to establish an ER stress activated and inhibited model. The data shown are representative of three independent experiments. (**B**) (bar = 20 µm) Representative confocal micrographs of GRP78 protein (green) infected with *B. suis* S2 (red) and ΔVceC (red) by added 0.5 µg/mL Tm or 1 µM 4-PBA before infection in GTCs. The blue represents cell nucleus. (**D**–**E**) CFUs of *B. suis* S2 (**C**) and ΔVceC (**D**) were determined in GTCs at 24 h post infection. The 0.5 µg/mL Tm or 1 µM 4-PBA was added before infection. Data represent the mean ± standard deviation from three independent experiments. * *p* < 0.05

To more directly assess the role of ER stress in *Brucella* survival and proliferation in GTCs, we explored whether ER stress altered by Tm or 4-PBA affects *B. suis* S2 or ΔVceC in GTCs. Increasing ER stress with 0.5 µg/mL Tm significantly increased ER stress marker GRP78 and CHOP expression (Figure 5A,B). In contrast, decreasing ER stress with 1 µM 4-PBA (Figure 5A,B) significantly inhibited GRP78 and CHOP protein expression. In addition, increasing ER stress with Tm significantly inhibited the proliferation of *B. suis* S2 or ΔVceC, and decreasing ER stress with 4-PBA enhanced the proliferation of *B. suis* S2 or ΔVceC (Figure 5B–E). This observation is consistent with other reports that *Brucella* proliferation in host cells is affected by apoptosis in infected cells. Furthermore, the proliferation of ΔVceC was significantly different compared with that of the *B. suis* S2 infection group under changing ER stress. The results reconfirmed that the deletion of VceC changes *Brucella*-mediated ER stress.

## 3. Discussion

The ER mediates biosynthesis, folding, and modification of secretory and transmembrane proteins as well as maintains of calcium homeostasis. Elevated physiological demand for protein folding can disrupt the ability of ER, leading to misfolded or unfolded protein accumulation in this organelle, a condition called ER stress. The unfolded protein response (UPR) is induced to restore ER homeostasis [18]. However, excessive stress to the ER triggers CHOP-induced apoptosis [20].

Our results indicated that the *B. suis* S2 T4SS-secreted protein VceC can trigger ER stress but inhibit CHOP-induced apoptosis. The manipulation of host cell death is a critical strategy of *Brucella* to maintain dissemination or long-term intracellular persistence [5,21]. Macrophage/monocytes infected with *Brucella suis* or *Brucella melitensis* stains inhibit the apoptosis pathways, whereas GTCs infected with *B. suis* S2 undergo apoptotic cell death mediated by ER stress [17,22,23]. T4SS is one of the key factors for *Brucella* intracellular survival and plays an essential role in the inhibition of host-cell death [24]. Inactivation of virB, which encodes the T4SS, in smooth *B. melitensis* prevented the cytotoxicity of *Brucella* for macrophages; in contrast, overexpression of virB enhances cytotoxic effects [24]. The VceC also mediates the cytotoxicity effect by translocation of this effector protein into macrophages, resulting in lysis of the host cells [11]. However, the cytotoxicity or lysis of *Brucella* for host cells resembles oncosis and nerosis, but not apoptosis [11]. In our studies, *B. suis* S2 infection induced caspase-9 protein expression in GTCs, but, interestingly, VceC mutants enhanced cleaved caspase-3 and CHOP protein expression and induced an increase in apoptosis in trophoblast cells. During ER stress, CHOP expression increases to activate downstream genes, leading to ER stress-induced apoptosis [25,26]. Martinon et al. [27] suggested that *Brucella* lipopolysaccharide (LPS) may sufficiently temper CHOP induction to avert apoptosis. These findings suggested that VceC may synergize with LPS to inhibit CHOP-induced apoptosis. Further work is needed to verify the interaction between VceC and LPS during *Brucella* infection. Therefore, our results demonstrated that VceC promoted intracellular persistence of *Brucella* infection, which may be related to decreased CHOP expression and inhibition of the ERS-induced apoptosis pathway.

UPR is known to support the *Brucella* intracellular life cycle within the host cell. First, the UPR mobilizes amino acid transport and supports lipid biogenesis. Second, the UPR regulates autophagy to providing more nutrients. Third, the UPR enhances the protein-folding capacity to suppress downstream apoptosis. Fourth, the UPR allows cells to cope with oxidative stresses. Finally, the UPR enables host cells to survive the disruption of the ER structure and function [16]. During *B. abortus* infection, the effector protein VceC interacts with the ER chaperone GRP78 and localizes to the ER, then disrupts the ER structure, and results in ER stress in HeLa cells [12]. Our previous study shows that inhibiting ER stress with 4-PBA increased the number of *B. suis* S2 CFUs in GTCs. Enhancing ER stress with Tm inhibited the proliferation of *B. suis* S2 in GTCs [17]. GRP78 plays an essential role in supporting *Brucella* replication in GTCs. Decreasing GRP78 expression inhibited *B. suis* S2 proliferation in GTCs by promoting ER stress-induced apoptosis [17]. Those findings are consistent with our result that the *VceC* mutant reduced the GRP78 expression and enhanced that of CHOP to promote ER stress-induced apoptosis. The replication of the VceC mutant was more sensitive under the different ER stress conditions in the GTC line after treatment with ER stress inhibitors 4-PBA or ER stress inducer Tm. Our studies indicate that effector protein VceC is essential for *Brucella* replication by interaction with GRP78 to inhibit the apoptosis of *B. suis* S2-infected GTCs.

ER stress transmembrane sensor IRE1 plays a pivotal role in *Brucella* replication. Previous studies suggested that *B. abortus* infection of macrophages or HeLa cells activates IRE1α pathway [16,28]. Yip1A, a host protein, is required for rBCV biogenesis and intracellular *B. abortus* replication through mediated IRE1 activation in HeLa cells [28]. De Jong et al. [12] demonstrated that IRE1-mediated UPR activation is activated by VceC. In our study, we confirmed that VceC mutant infection did not activate the IRE1 pathway in GTCs. VceC mutant infection did not affect the *B. suis* S2 CFU in GTCs. Our results are consistent with the study that IRE1 knockdown in bone marrow-derived macrophages (BMDM) or decreasing phosphoIRE1α and IRE1α in GTCs did not affect the number of *Brucella* bacteria at 24 h post infection [17,28]. An in vivo study suggested that infection with the *B. abortus* wild type and the VceC mutant resulted in similar numbers in the placenta of mice [13]. Taken together, we speculated that more effector protein synergies exist with VceC to activate the IRE1 pathway of UPR to support the *Brucella* replication in host cells, since inactivation of VceC alone did not affect the replication competence of *Brucella* [12].

In summary, the results demonstrated that *B. suis* S2 T4SS effector protein VceC decreased CHOP expression, interacted with GRP78 to inhibit apoptosis, and mediated the IER1 pathway of UPR to support *B. suis* S2 replication in GTCs. Our findings also suggested that more effector proteins should exist in synergy with VceC to support persistent *Brucella* intracellular infection. The present work provides new insights for understanding the mechanism of VceC in the establishment of chronic *Brucella* infection.

## 4. Materials and Methods

### 4.1. Bacterial Strains

The bacterial strains used in this study were smooth attenuated virulent *Brucella suis* vaccine strain 2 (*B. suis* S2, CVCC70502), and they were obtained from the Chinese veterinary culture collection center (Beijing, China). The *B. suis* S2 were grown in tryptic soy broth (TSB; Takana) and tryptic soy ager (TSA; Takana). Goat trophoblast cells (GTCs) were immortalized by transfection with human telomerase reverse transcriptase (hTERT); these cells were provided by Dewen Tong (Northwest A&F University, Yangling, Shaanxi, China). For infection of GTCs, the bacteria were collected by centrifugation at 6000× *g* for 10 min at 4 °C and washed three times with 15 mL of phosphate-buffered saline (PBS). The number of *B. suis* S2 were counted by plating on TSA. *Escherichia coli* strain DH5α (takana) was cultured in Luria-Bertani (LB) medium. When appropriate, 50 μg/mL gentamicin or ampicillin were respectively added. Plasmid PUC19 was purchased from Takana.

### 4.2. Construction of the Mutant Strain ΔVceC

ΔVceC was constructed as described previously [29]. Briefly, primers were designed using the sequence *B. suis* S2 genome and plasmid PBBR1MCS-5. The 896 bp *Vce*C upstream fragment, 964 bp *Vce*C downstream fragment, and 751 bp gentamicin fragment were obtained in three independent PCR reactions using primer STAR Max Mix with primer pairs *Vce*C-UF: ctgcag (*Pst*1) TCGGAAGCGAGCACCTGA; *Vce*C-UR: tctaga (*Xba*I) GCGGATACCCTCTTACACTATAAAC; *Vce*C-DF: gagctc (*Sac*I) CCAAGGGAGAAACCCGCA; *Vce*C-DR: gaattc (*EcoR*I) CGTGTTCACAACCGATAAGG; G-F tctaga (*Xba*I) TTGACATAAGCCTGTTCGGTTCGTA; G-R: gagctc (*Sac*I) TTAGGTGGCGGTACTTGGGTCGATA. After purification by gel extraction, the three fragments were cloned into PMD19T-simple, then digested with *Pst*I and *Xba*I; *Sac*I and *EcoR*I; and *Xba*I and *Sac*I sequentially, and then subcloned into the *Pst*I- and *EcoR*I-digested PUC19 plasmid. The recombinant plasmid with the correct sequence was designated PUC19-VceC. Then this plasmid was electroporated into *B. suis* S2, where it is incapable of autonomous replication. The potential GntR deletion mutant was selected by plating on TSA-containing gentamicin, which was then verified by PCR with primer pairs VceC-F: CTTCTCATTGGCAAGCACTTC and VceC-R: GCATCATTCGCCGTTTCA. The mutant strain was called ΔVceC.

### 4.3. Cell Infection Assay

The process of the *B. suis* S2 infection assays was carried out as described previously. Briefly, GTCs were seeded in 6-well plates (5 × 10^5^ cells per well) or in 24-well plates (1 × 10^5^ cells per well) and were infected with *B. suis* S2 or ΔVceC at a multiplicity of infection of 100:1. After 4 h of incubation at 37 °C with 5% CO_2_ atmosphere, GTCs were washed three times with PBS and then further cultured with cell culture medium containing 50 µg/mL kanamycin to eliminate *B. suis* S2 or ΔVceC adhering to the GTCs and in the culture medium. After 1 h, the GTCs were washed three times with PBS and were further cultured with cell culture medium containing 25 µg/mL kanamycin to avert continuous infection. This point in time was considered 0 h and the time point of treatment with Tm (ER stress activator) and 4-PBA (ER stress antagonist). The cells were collected, and relevant experiments were performed at specific times (0, 6, 12, 24, and 48 h).

For intracellular survival assays, cells were seeded in 24-well plates prior to infection. Then, cells were infected with *B. suis* S2 or ΔVceC as described. At different times following infection, wells of infected cells were washed three times with PBS and lysed with 0.5% Triton X-100 in PBS for 10 min. The lysates were serial diluted in PBS and plated onto TSA for 72 h to determine the colony-forming units (CFUs).

For adherence, cells were seeded in 24-well plates prior to infection. Then, cells were infected with *B. suis* S2 or ΔVceC as described. After 1 h of incubation at 37 °C with 5% CO_2_ atmosphere, wells of infected cells were washed three times with PBS and lysed with 0.5% Triton X-100 in PBS for 10 min. Next, the lysates were serial diluted in PBS and plated onto TSA for 72 h to determine the colony-forming units (CFUs).

### 4.4. Western Blot Analysis

GTCs were harvested in a tube after infection at 0, 12, 24, and 48 h, and then lysed on ice for 30–45 min in lysis buffer. The supernatant was obtained by centrifugation for 15 min at 14,000 rpm at 4 °C. The protein concentration was determined by the Bicinchoninic acid (BCA) assay. Total cellular protein was extracted with 5 × sodium dodecyl sulfate polyacrylamide gel electrophoresis (SDS-PAGE) loading buffer after boiling for 5 min in water. Samples were electrophoresed on a 12% polyacrylamide gel for SDS-PAGE. The gels were then electro-transferred onto polyvinylidene fluoride (PVDF) membranes. The membranes were blocked for 1 h in Tris-buffered saline containing 0.5% Tween-20 (TBST) with 5%–10% skimmed milk at room temperature and then incubated overnight at 4 °C in blocking solution containing GRP78 (Abcam, 1:1000 dilution), CHOP (Abcam, 1:1000 dilution), ATF-6 (Abcam, 1:1000 dilution), phosphoIRE1 (Abcam, 1:1000 dilution), phosphoeIF2α (Abcam, 1:1000 dilution), caspase-3 (proteintech, 1:1000 dilution), caspase-9 (proteintech, 1:1000 dilution), and anti-β-actin (Tianjin Sungene Biotech Co, 1:1000 dilution). The membranes were washed five times with TBST for 5 min and then incubated for 1 h with the corresponding secondary antibody conjugated to horse radish peroxidase (HRP) (1:5000, Zhongshan Golden Bridge Biotechnology, Nanjing, China). Finally, the membranes were washed five times in TBST for 5 min. The blots were visualized using the Gel Image System (Tannon, Biotech, Shanghai, China).

### 4.5. Immunofluorescence Assay

GTCs were seeded in 24-well plates and were infected with *B. suis* S2 and ΔVceC at 100 MOI. At 0 and 24 h post infection, infected cells were washed twice with PBS and then fixed with 4% paraformaldehyde at room temperature for 30 min. After three washes with PBS, cells were incubated with PBS containing 0.25% Triton X-100 at room temperature for 20 min. After three washes with PBS, goat anti-brucella polyclonal antibody (1:100 dilution), rabbit anti-GRP78 monoclonal antibody (1:200 dilution), and mouse anti-LAMP-1 monoclonal antibody (1:200 dilution) were used as the primary antibody. Donkey anti-goat alexa fluor 555, donkey anti-mouse alexa fluor 488, and donkey anti-rabbit alexa fluor 488 were used as the secondary antibody at 1:200 dilutions. Next, coverslips were mounted on glass slides, and cells were observed under a microscope. Assays were performed in triplicate.

### 4.6. Statistical Analysis

Statistical analysis was performed using Graphpad Prism software 6 (GraphPad software Inc., La Jolla, CA, USA). Statistical significance was determined using two-way ANOVA or one-way ANOVA. P values less than 0.05 were considered statistically significant.

## Figures and Tables

**Table 1:** Results of Annexin V-FITC/PI staining for cell apoptosis after *B. suis* S2 and ΔVceC infection at 12 h.

Group	Normal Cells	The Early Apoptotic Cells (%)	The Late Apoptotic Cells (%)
Control	85.05 ± 0.63	1.77 ± 0.23	3.59 ± 0.25
*B. suis S2*-infected	84.45 ± 4.03	1.23 ± 0.34	3.96 ± 0.49
ΔVceC-infected	84.55 ± 1.06	3.04 ± 0.10 *	4.08 ± 0.74

Data represent the means ± standard deviations from three replicates. The asterisks (*) represent significant differences (*p* < 0.05) of the cell cycle distribution in GTCs infected by ΔVceC compared to that in *B. suis* S2-infected cells.

**Table 2:** Results of Annexin V-FITC/PI staining for cell apoptosis after *B. suis* S2 and ΔVceC infection at 48 h.

Group	Normal Cells	The Early Apoptotic Cells (%)	The Late Apoptotic Cells (%)
Control	75.20 ± 1.41	5.15 ± 2.46	8.01 ± 4.51
*B. suis S2-*infected	79.50 ± 3.68	5.25 ± 0.71	6.18 ± 0.51
ΔVceC-infected	72.85 ± 2.47	8.96 ± 0.99 *	6.37 ± 0.98

Data represent the means ± standard deviations from three replicates. The asterisks (*) represent significant differences (*p* < 0.05) of the cell cycle distribution in GTCs infected by ΔVceC compared to that in *B. suis* S2-infected cells.

## Supplementary Materials

Supplementary materials can be found at https://www.mdpi.com/1422-0067/20/17/4104/s1. Supplementary Figure 1: Results of cell apoptosis after B. suis S2 and ΔVceC infection at 12 h and 48 h.
