# Supplementary material for: VceC Mediated IRE1 Pathway and Inhibited CHOP-induced Apoptosis to Support Brucella Replication in Goat Trophoblast Cells
Source: Int J Mol Sci. 2019 Aug 22;20(17):4104. doi: 10.3390/ijms20174104 (PMC6747397; doi:10.3390/ijms20174104)
Supplement: Supplementary file 1 [file ijms-20-04104-s001.pdf]

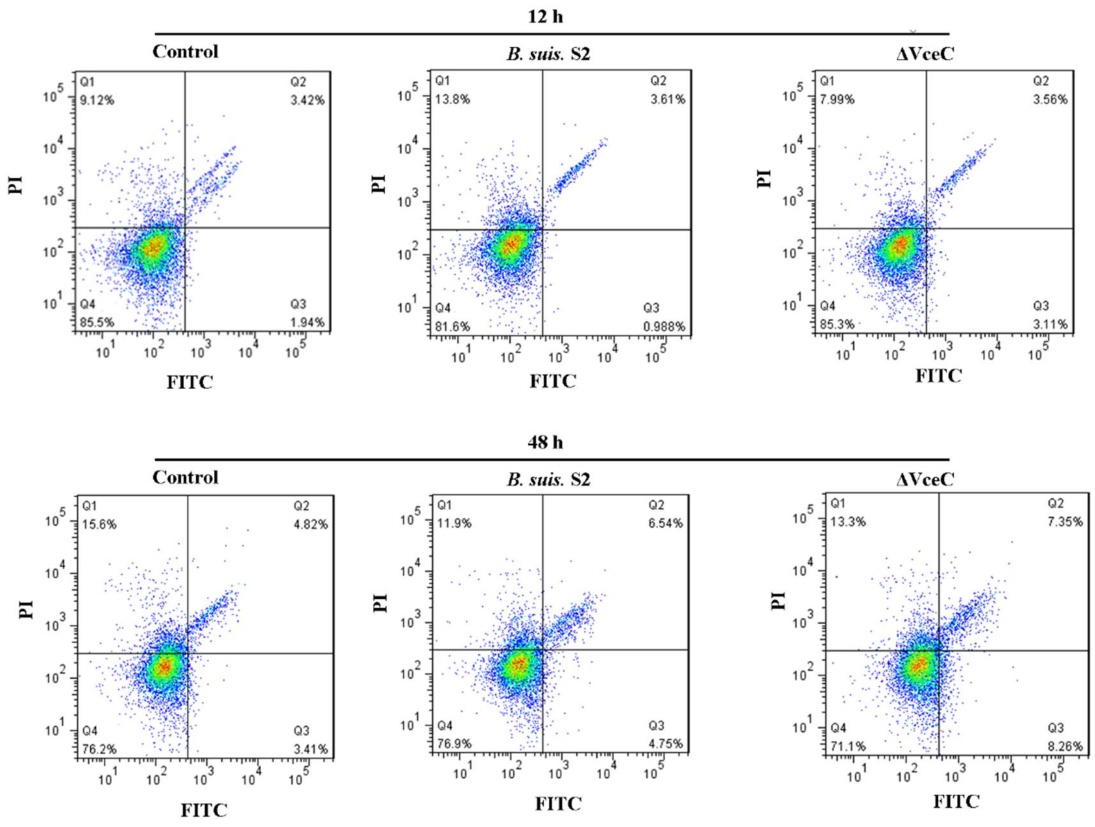

Supplementary figure 1 Results of cell apoptosis after *B.suis*.S2 and ΔVceC infection at 12 h and 48 h. To assess cell death in vitro, GTCs cells were infected with *B.suis*.S2 or ΔVceC for 12 h and 48 h; the cells were then subjected to Annexin V-FITC/PI staining and analyzed by flow cytometry. All experiments were repeated three times with similar results.
